# Insights of Severe Acute Respiratory Syndrome Coronavirus (SARS-CoV-2) pandemic: a current review

**DOI:** 10.1186/s12575-020-00141-5

**Published:** 2021-02-01

**Authors:** Jyoti Choudhary, Shrivardhan Dheeman, Vipin Sharma, Prashant Katiyar, Santosh Kumar Karn, Manoj Kumar Sarangi, Ankit Kumar Chauhan, Gaurav Verma, Nitin Baliyan

**Affiliations:** 1 Department of Microbiology, Chinmaya Degree College (Hemwati Nandan Bahuguna Garhwal University, Srinagar, Garhwal, Uttarakhand), Haridwar, Uttarakhand 249401 India; 2 Department of Botany and Microbiology, Gurukula Kangri Deemed to be University, Haridwar, Uttarakhand 249404 India; 3 Department of Microbiology, School of Life Sciences, Sardar Bhagwan Singh University, Dehradun, Uttarakhand 248161 India; 4 Department of Pharmaceuticals Sciences, Faculty of Ayurvedic and Medicinal Sciences, Gurukula Kangri Deemed to be University, Haridwar, Uttarakhand 249404 India; 5 Deaprtment of Biotechnology and Biochemistry, School of Life Sciences, Sardar Bhagwan Singh University, Dehradun, Uttarakhand 248161 India; 6 Department of Pharmaceutical Sciences, School of Pharmaceutical Sciences and Technology, Sardar Bhagwan Singh University, Dehradun, Uttarakhand 248161 India; 7 Atal Bihari Vajpayee Institute of Medical Sciences and Dr. Ram Manohar Lohia Hospital, New Delhi, 110001 India; 8 Deaprtment of Microbiology, Shri Dev Suman Subharti Medical College, Ras Bihari Bose Subharti University, Dehradun, Uttarakhand 248001 India

**Keywords:** COVID-19, Coronavirus, ACE-2 receptor, Drug repurposing

## Abstract

COVID-19, a pandemic of the 21st century caused by novel coronavirus SARS-CoV-2 was originated from China and shallowed world economy and human resource. The medical cures via herbal treatments, antiviral drugs, and vaccines still in progress, and studying rigorously. SARS-CoV-2 is more virulent than its ancestors due to evolution in the spike protein(s), mediates viral attachment to the host’s membranes. The SARS-CoV-2 receptor-binding spike domain associates itself with human angiotensin-converting enzyme 2 (ACE-2) receptors. It causes respiratory ailments with irregularities in the hepatic, nervous, and gastrointestinal systems, as reported in humans suffering from COVID-19 and reviewed in the present article. There are several approaches, have been put forward by many countries under the world health organization (WHO) recommendations and some trial drugs were introduced for possible treatment of COVID-19, such as Lopinavir or Ritonavir, Arbidol, Chloroquine (CQ), Hydroxychloroquine (HCQ) and most important Remdesivir including other like Tocilizumab, Oritavancin, Chlorpromazine, Azithromycin, Baricitinib, etc. RT-PCR is the only and early detection test available besides the rapid test kit (serodiagnosis) used by a few countries due to unreasonable causes. Development of vaccine by several leader of pharmaceutical groups still under trial or waiting for approval for mass inoculation. Management strategies have been evolved by the recommendations of WHO, specifically important to control COVID-19 situations, in the pandemic era. This review will provide a comprehensive collection of studies to support future research and enhancement in our wisdom to combat COVID-19 pandemic and to serve humanity.

## Highlights

- State-of-status on a pandemic of COVID-19
- Discussing the controversies of origin of SARS-CoV-2
- Overall account to understand SARS-CoV-2 at the molecular level
- Accounting pathogenic attacks of the virus in human cells
- Citing development of vaccines, possible drug and target sites for the treatment

## Introduction

The emergence of pandemics brings dares to control their harmful impacts on human health worldwide. In this catastrophic situation ofa novel infectious disease caused by a novel SARS-CoV-2,there is a flood of research papers on various aspects and impacts. The theories of virus origin have shifted the quantum towards controversies. Initially, it was recognized as a beta coronavirus and identified as able to cause a pandemic. Covering 75–80% similarity in genetic sequence to SARS-CoV, this virus embarked itself on a novel lineage with new and evolved or recombined spike proteins. However, the attention of a microbiologist or virologist is to summarize its pathogenesis and cure avenues. It is crucial to understand the mechanism of the viral evasion, pathogenesis, and apply our wisdom to find novel drugs or repurposing the existing ones.

Through back in the memories of virology, the first human coronavirus was isolated from the human’s nasal cavity by Tyrrell and Bynoe [182], as a common virus not having pathogenic features. However, all the variants of coronavirus species present in humans have originated from bats [103]. Li et al. [109] proposed a hypothesis of ancestral recombination among different coronaviruses infecting bats and pangolinsas an indicationof SARS-CoV-2 evolution and infection in humans via food chain including other modes, like air-borne transmission, and still under study. The respiratory droplets served in transmission, produced from cough and sneeze of an infected individual, and if inhaled can settle in the lungs, nasal mucosa to cause respiratory illness. Raj et al. [140, 141] has been pointed out an epidemic spread with a very high reproduction rate (R_0_ 4.71) in the initial, but later observed to declined to 2.08, suggests a gradual decline in infection with time. The World Health Organization (WHO) has named this disease as‘COVID-19’ and classified into mild, moderate, severe, and critical categories [193]. Like other viral infections, a typical pattern of IgM and IgG production was observed by antibody profiling. The antigen presentation can also be affected by this novel coronavirus. Therefore, destroying the immune evasion of SARS-CoV-2 is imperative to find a therapeutic approach and specific drug development. There is no approved drug therapy for SARS, MERS, or even for COVID-19 infection in the current scenario, also lack clinical trial data on COVID-19 treatment makes the condition even worse. Various herbal treatments such as papain-like protease (PL^pro^), 3C-like protease (3CL^pro^) and alternative therapeutic drugs such as Lopinaviror, Ritonavir (HIV infections), Arbidol (influenza infection), Remdesivir (Ebola virus), Chloroquine and Hydroxychloroquine (Malaria), Tocilizumab (CRS secondary to CAR [chimeric antigen receptor] T-cell therapy), Teicoplanin (MERS-CoV), Glucocorticoids (pneumonia such as SARS and MERS), Baricitinib (Rheumatoid Arthritis) are in the active research trialsfor the treatment of COVID-19. Clinical data rigorously evaluated or refuted in high-quality randomized trials, particularly given that for COVID-19 has no proven safe and effective treatments yet, beside remdesivir accepted by many countries of Asia and including most severely affected Unites States of America [62]. In recent use of cc-miRNAs is advantageous to suppress infections can be an approach to control the virus in host cells during its replication [142]. Raj et al. [140, 141] reviewed notably on the mechanism (s) of virus replication, mode of infection in humans, and development of novel vaccines or drugs for the eradication and prevention.

This review figures out the status of knowledge on COVID-19 and SARS-CoV-2. In the flood of research papers, it combines all advancement in the box full of concise information. The review presents the origin of the virus, its background, pathogenicity, clinical manifestations, possible ways of treatment, and management to date. This review will be beneficial to undergraduates to the research community to have a glance of present knowledge. This further advances our understanding of SARS-CoV-2 and its impact on the present research scenario.

## Origin of SARS-CoV-2 and epidemiology

The two scenarios for the origin of SARS-CoV-2 were proposed by Andersen et al. [2] that it may be borne by (i) natural selection in an animal host before zoonotic transfer; and (ii) natural selection in humans following a zoonotic transfer. As Zhu et al. [227] have published, the first pneumonic syndrome was originated from a market where sea-food and wet animals were traded. The first report of novel pneumonia (COVID-19) was in Wuhan city of Hubei province in China, occurred in late December 2019, although, the reflective analyses have identified patients with symptoms of onset short-breathing as on early December 1, 2020 [225]. Later, Tang et al. [176] suggested that SARS-CoV-2 is a new variant of coronavirus probably due to the mutation rathera recombination. Studies classify it into S and L type based on virulence and proved them originated as a result of natural selection due to the change in functional sites in the receptor-binding domain (RBD) of the SARS-CoV-2 spike and SARS-CoVs viruses from pangolin. Andersen et al. [2] explained that SARS-CoV-2 is not a purposefully manipulated virus based on molecular sequencing data and scientific shreds of evidence were credited to provide a conclusion that it is impossible to prove or disprove the other theories in the current context. There is the plausibility of balance towards the evidence of origin by one hypothesis over the other based on ongoing studies of pneumonia in humans and other animals. An article changed the quantum by adding pieces of evidence into the direction of recombination from the natural selection as suggesting strong purifying selection around the receptor-binding motif (RBM) in the spike gene and other genes among bat, pangolin and human coronaviruses, indicating similar strong evolutionary constraints in different host species [109]. Li et al. [109] gave a hypothesis that this, and/or other ancestral recombination events between viruses infecting bats and pangolins, may have a key role in the evolution of the strain that leads to the introduction of SARS-CoV-2 into humans. The proximity of animals of different species in a wet market setting increased the potential of cross-species spillover infections, by enabling recombination between more distant coronaviruses and the emergence of recombinants with novel phenotypes. As per the epidemiological timeline by WHO (https://www.who.int/emergencies/diseases/novel-coronavirus-2019/interactive-timeline#!) on Jan 9, 2020, reports says Chinese authorities have determined that the outbreak is caused by a novel coronavirus. Following on Jan 24, 2020, they held an informal consultation on the prioritization of candidate therapeutic agents for use in novel coronavirus infection. On Feb 11, 2020, they announced that the disease caused by the novel coronavirus would be named COVID-19. Following best practices, the name of the disease was chosen to avoid inaccuracy and stigma and therefore did not refer to a geographical location, an animal, an individual, or a group of people. In the present state, over 69,143,017confirmed COVID-19 cases and 1,576,516 deaths were reported to WHO for the week ending 11 December 2020 [198]. The highest number of cases was alone observed in America and India, where 230,852 newly cases were reported in a 24 h (as on 12 December 2020), in America and toll of new cases is nearly similar in India i.e., 29,398 cases.

## Background of SARS-CoV-2

The coronavirus is an enveloped, (+)ssRNA viruses, important to both medical and veterinary sciences. Coronavirus is distributed among mammals and birds and known that it causes respiratory, enteric, and neurologic diseases [97]. However, all the variants of coronaviruses species present in humans have been originated from the bats [10, 20]. They are membersof order Nidovirales, family Coronaviridae [43], subfamily Orthocoronavirinae, genus Betacoronavirus, and subgenus Sarbecovirus [227]. There are four genera α, β, γ, and δ represents coronaviruses. Where α and β are pathogenic to mammals and γ and δ can cause serious illnesses in birds. As mentioned earlier, coronavirus has been placed in order nidovirales, the meaning of Nido in Latin “Nest” thus its order name itself explains- these viruses nested in 3 special features. First,expression of enzyme, replicase polyprotein via ribosomal frameshifting. Second,unique enzymatic activities among the replicase protein products, and last a virion membrane envelope with multi-spanning integral membrane protein [43].

Different six strains of coronavirus within the α and β groups [131, 175] are pathogenic to humans. These six coronaviruses are named as coronavirus-229E (HCoV-229E), HCoV-OC43, HCoV-HKU1, Severe Acute Respiratory Syndrome - coronavirus (SARS-CoV), HCoV-NL63 and Middle East respiratory virus Coronavirus (MERS-CoV) [215]. These viruses are already known for their association with both the upper and lower respiratory tract and causing related diseasesin humans. Among these six human-related coronaviruses, SARS-CoV, and MERS CoV are important viruses causing serious respiratory illnesses;besides, four others cause mild diseases [228]. The present outbreak of SARS-CoV-2 is a third major spillover animal-related coronavirus to humans in the last two decades which causes a major pandemic.

## Genome organizationand molecular evolution

**Fig. 1:**
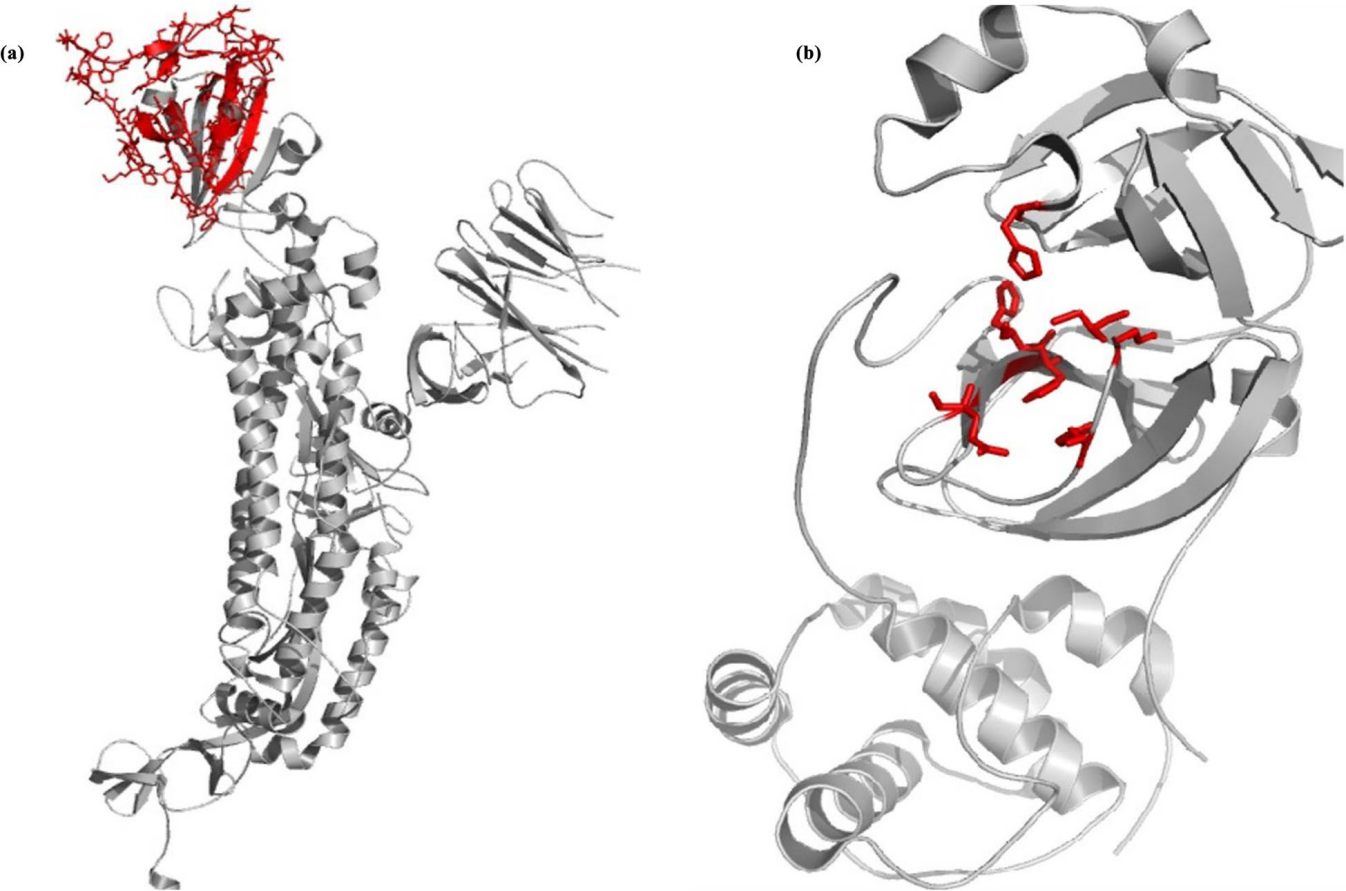
**a** The crystal structure of SARS-CoV-2 spike protein. The human ACE-2 receptor binding domain of spike protein is shown in red color (PDB ID: 6VSB) (**b**) 3D structure of SARS-CoV-2 main protease. Residues in the active site (His-41, Gly-143, Phe-140, and Glu-166) are shown in red color (PDB ID: 6 LU7) obtained from protein data bank (http://www.rcsb.org/pdb/)

Coronaviruses are enveloped viruses with a positive-sense single-stranded RNA genome (26–32 kb) which is largest among all RNA viruses and gives flexibility in harboring and rearrangement of their genes [172]. The genomic analysis of SARS-CoV-2 showed 75–80% similarity with SARS-COV [135, 227] and revealed the presence of coding sequence for structuralas well as non-structural proteins. These incorporate spike glycoproteins, responsible for attachment in the host cell, RNA dependent RNA polymerase (RdRp), and papain-like proteases (PL^pro^) [144]. The attack is also activated by priming response by host’s serine protease (TMPRSS211) [155]. When the virus invades a hostcell, it releasesits single-stranded RNA into the host cell and multiply with the assistance of host cell protein-synthesizing hormone and produces enormous amounts of the viral genome. Till now, bat presumed as the prominent host of SARS-CoV-2and an unidentified intermediate host may also be involved [135]. The tomographic study of intracellular structures involved in virus replication and assembly showed that Coronavirus is assembled completely at the pre-Golgi compartment membrane [90]. SARS-CoV-2genome possesses 14 ORF(s) which encodes 27 protein. *Orf1ab *is identified as the largest gene [204]. First ORF (ORF1a/b) encodes a two-thirds viral RNA, which later translated into two polyproteins of replicas, pp1a and pp1ab include 16 nonstructural proteins (nsps) as a viral replicase transcriptase complex. A very few of coronaviruses encode a hemagglutinin esterase (HE). Several other accessory proteins seem to be important for pathogenesis, but all are not characterized for their functionalities. A pre-published scientific contribution of Gordon et al. [59–61] highlighted SARS-CoV-2 proteins and human proteins for host-viral interaction with a range of functions including DNA replication (Nsp1), epigenetic and gene expression regulators (Nsp5, Nsp8, Nsp13, E), vesicle trafficking (Nsp6, Nsp7, Nsp10, Nsp13, Nsp15, Orf3a, E, M, Orf8), lipid modification (Spike), RNA processing and regulation (Nsp8, N), ubiquitin ligases (Orf10), signaling (Nsp8, Nsp13, N, Orf9b), nuclear transport machinery (Nsp9, Nsp15, Orf6), cytoskeleton (Nsp1, Nsp13), mitochondria (Nsp4, Nsp8, Orf9c), and extracellular matrix (Nsp9). These nsps reorganize host’s rough endoplasmic reticulum (RER) membranes for its origination in a double-membrane vesicle envelope [90]. Research of Khailany et al. [88] stated differentiation of single point transformations from nonsynonymous substitutions in nsps 4, 5 10 12 14 and 16 as the cause of ORF1b nsp 12 14 and 16 proteins (cistrons) and distortion of ORF1a proteins nsps 4, 5, and 10 coding sequence of ORF1ab.The 5′ terminus contains the *Orf1ab *and 3′ terminus encode structural gene for four structural proteins S, E, M, N, and 6 accessory proteins (Fig. 1). The additional proteins are encoded by ORF3a, ORF6, ORF7a, ORF7b, and ORF8 genes [88]. SARS and SARS-CoV-2are quite similar based on amino acid level except at certain points [115] such as ORF8 a protein (present in SARS-CoV but absent in SARS-CoV-2) while, ORF8b protein of SARS-CoV-2is larger than SARS-CoV. Similarly, protein 3b is quite shorter in SARS-CoV-2 than that ofSARS-CoV [205].

**Fig. 2:**
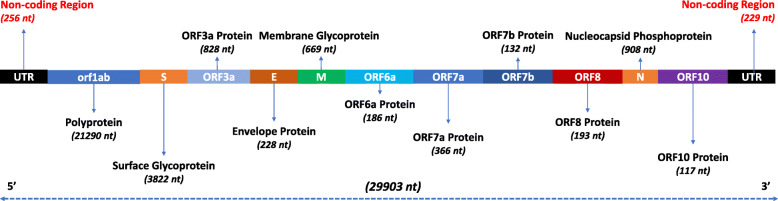
Complete genomic structure of the novel SARS-CoV-2 virus (29,903 nucleotides) (Credit: [88])

In our study, COVID-19 (309 residues are built by the aid of the automated homology modeling, Swiss Model, web server using the crystal structure) possesses protease in complex with inhibitor n3 (PDB ID: 6 LU7, chain A) as a homolog. The model has a very high (98.00%) sequence identity to the template. Refinement was carried out in PHENIX 1.17.1_3660. The Ramachandran plot shows 100% of the residues in the allowed regions, 97.00% in the most favored region (Fig. 2) suggesting molecular identity of SARS-CoV-2 with its ancestors.

## Structural proteins and accessory proteins

**Fig. 3:**
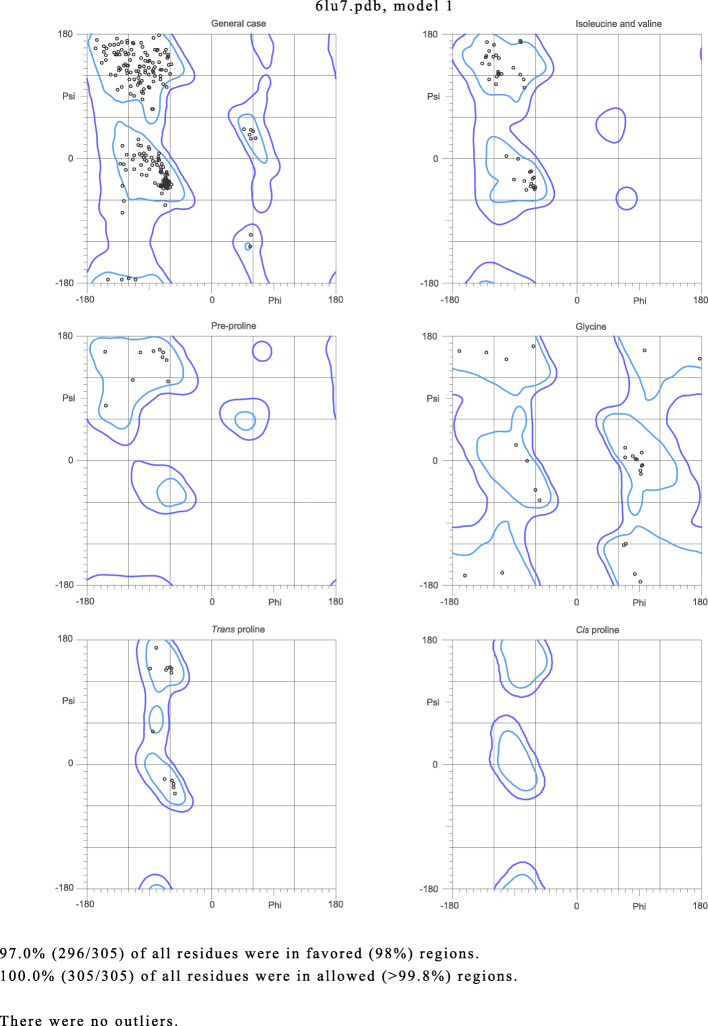
Ramachandran plot for COVID- 19 using the crystal structure of COVID-19 main protease in complex w inhibitor n3 (PDB ID: 6 LU7, chain A) (http://kinemage.biochem.duke.edu) [32]

SARS-CoV-2 encodes more than one dozen proteins, some of which are essential for viral entry and replication, and most significant are papain-like protease (PLpro), 3C-like protease (3CLpro) and spike protein. Coronavirus PLpro processes the viral polypeptide onto functional proteins and is also a deubiquitinating enzyme that dampens the host anti-viral response by hijacking the ubiquitin (Ub) system. It has been reported that SARS PLpro cleaves ISG15 (a two-domain Ub-like protein) and Lys48-linked polyUb chains and releases diUbLys48 products [11, 81]. SARS-3CLpro is a cysteine protease indispensable to the viral life cycle [122]. Coronavirus spike protein uses angiotensin-converting enzyme 2 as a receptor to help the virus enter cells. Coronaviruses contain 3 envelope protein i.e., spike (S) protein, membrane (M) protein, envelope (E) protein, and nucleocapsid (N) protein. Spike proteins are the glycoproteins [8, 18] which mediate host cell and virus binding followed by induction of human immune system, harvesting byscientists for vaccine development [137]. Wrapp et al. [203] determined the first Cryo-EM structure of novel coronavirus spike (S) protein. The S protein accommodates furin-cleavage sites between S1 and S2 processing during the biogenesis and differs from SARS-CoV and other related coronaviruses [188]. Depending on the species, coronaviruses having separate domains of the S1 subunit recognizes different receptors for host-cell entry [77]. The S1 is the most divergent and mutable region whileS2 is conserved [54, 132]. The S1 subunit is the N-terminal space that binds with the host’s membrane receptors through its receptor-binding domain (RBD) and the S2 is the C-terminal space [48, 219]. The RBD of the S1 subunit is thus, liable for the zoonotic transmission, identification of host cell, and attack (Fig. 3). In some coronaviruses, small projections also observed between the spikes are short in length, called hemagglutinin-esterase (HE) protein [66].

The M protein is an abundant glycoprotein in the coronavirus responsible for the shape of the virus [124], and important for viral assembly in the host’s cell in the lytic phase [1]. Also, M protein is an outspanning protein, theamino-terminal domainsare in the endoplasmic reticulum [113]. The 25 amino acids peptide of M protein is highlyconserved while ectodomain is least [35]. Besides,M protein interacts with ribonucleotide protein (RNP) and S glycoproteins onbudding sitesto facilitate viral particle assembly; as earlier saidby Escors et al. [45, 46] and de Haan et al. [33]. The M and N protein having a role in genome packagingwere found crucial identification at molecular level. M protein provides the signal for the genome packaging with the interaction of RNA [123] while N proteins bind to the genome and form the nucleocapsid of coronavirus [34] and support to the viral replication [118]. It frames a network of M-N interaction compelling to exclude host membranesprotein [1, 35]. N protein ranged from 43 to 50 kDa and a major component of the helical nucleocapsid of the coronavirus [99, 167]. N protein is primarily involved in the nucleocapsid formation, replication cycle of the virus, and interferes with the host cellular response against the virus [118]. The presence of the N protein at the endoplasmic reticulum (ER)-Golgi region during viral infection proved its function in the assembly and budding of the virus [149, 158, 180].

The E protein is small (8.4–12 kDa) and recondite proteins of virions, which are extremely divergent among different groups of coronaviruses (E protein express abundantly during the replication of the virus in host though their presence in the virion envelope is minor compared to others [186]. These proteins are mainly found in the ER-Golgi intermediate compartment where they mediate the assembly, budding and intracellular trafficking of infectious virions [110, 126, 218].

## Pathogenicity and pathogenesis

Due to highly evolved and novelpathophysiology and virulence mechanisms, coronavirusesbecome a major cause of emerging respiratory disease outbreak, COVID-19. Coronavirus can directly enter at the cell surface after binding to the receptor or after internalization via endocytosis in the endosomal compartment [73]. The viral particles assemble in the Golgi, accumulate in dilated vesicles then transported and secreted to the cell surface, where they are released by exocytosis [108].

First and foremost, spike (S) proteins help in the attachment of coronavirus with the specific receptor play a significantrole in host cell invasion [83]. However, attachment influences with conformational changes in the virus spike protein. These conformational changes arise due to receptor binding and other activities that trigger receptors binding such as pH acidification or proteolytic activation. But novel coronaviruses modified their spike proteins with time which may be the cause of the diversity of triggers for the entry and fusion of the virus with host membrane [9, 108].

The receptor-binding domain of the S protein strengthens the affinity of S protein with its receptor which enhances the pathogenicity of SARS-CoV-2, which is like SARS-CoV [189]. SARS-CoV can fuse directly at the cell surface in the presence of relevant exogenous protease. It is believed that this route of entry is 100 to 1000-fold more efficient than the endosomal pathway [117].

The pathogenesis of SARS-CoV-2 is still mysterious and poorly understood. Scientific pieces of evidence based on genomic analysis suggested SARS-CoV-2 uses the angiotensin-converting enzyme 2 (ACE2) human receptor-like SARS-CoV [205]. A unique two-step furin activation in MERS-CoV for membrane fusion suggests strong shreds of evidence of similar mechanisms, adopted by SARS-CoV-2 [121]. The endocytosis entry of SARS-CoV has been reported either a clathrin-dependent and -independent mechanism [91]. The virus, after entry in the host’s cell releases its genome for the translation of two polyproteins and structural proteins [136, 165]. Then, viral particles matureinto the endoplasmic reticulum-Golgi intermediate compartment (ERGIC). Finally, the virus particles in the vesicles, fuse with the plasma membrane released out with envelopes and spikes [38].

The target cells of infection, its antigen is presented to the antigen presentation cells (APC) and recognized by virus-specific cytotoxic T lymphocytes (CTLs) via a presentation by major histocompatibility complex (MHC). Unfortunately, the understanding of the antigen presentation of SARS-CoV-2 is still in infancy. In the case of SARS-CoV, antigen presentation depends on MHC I and MHC II contribute in the presentation as complimentary [192]. However, during MERS-CoV infections, the MHC II molecules were found associated with the susceptibility to MERS-CoV infections [68]. Like other viral infections, a typical pattern of IgM and IgG production is evidenced by antibody profiling. The IgG antibody remains for a long time irrespective of IgM antibodies, which disappear at early phases and indicates a protective role of IgG antibody [105]. The SARS-specific IgG antibodies are primarily produced against S-protein and N-protein. The recent study illustrated the majority of CD4+ and CD8+ T cells circulating in the peripheral blood of COVID-19 patients reduces significantly, besides its excessive activation evidenced a high ratio of CD4 (3.47%) and CD38 (39.4%) cells [209]. These findings may provide valuable information for the understanding of immunological responses and rational to design vaccines against SARS-CoV-2. The antigen presentation can also be affected by this novel coronavirus. Therefore, destroying the immune evasion of SARS-CoV-2 is imperative in its treatment and specific drug development.

## Clinical features

Coronaviruses cause diseases of the respiratory, hepatic, nervous system, and gastrointestinal systems in humans. COVID-19 case reports showed a patient at 5 days of fever presented with a cough, coarse breathing sounds of both lungs, and a body temperature of 39 °C. SARS-CoV-2 shows the wide spectrum of clinical features that range from asymptomatic, symptomatic to multi-organ failure [25]. Based on the severity, the disease may be classified into mild, moderate, severe, and critical [195]. Mild symptoms include dry cough, mild fever, nasal congestion, sore throat, headache, and muscle pain, moderate disease includes shortness of breath, and tachypnea while severe disease shows the respiratory distress, respiratory failure, cardiac injury, septic shock, or multiple organ failure [28, 75, 76].

Laboratory features of patients infected with COVID-19 showed leukocytosis with abnormal respiratory findings and increased levels of plasma pro-inflammatory cytokines [102]. Patient with COVID-19 infection showed the high level of cytokines and some chemokines such as IL1-β, IL1RA, IL7, IL8, IL9, IL10, basic FGF2, GCSF, GMCSF, IFNγ, IP10, MCP1, MIP1α, MIP1β, PDGFB, TNFα, and VEGFA. In severe cases of COVID-19 infection high level of pro-inflammatory cytokines was also observed including IL2, IL7, IL10, GCSF, IP10, MCP1, MIP1α, and TNFα which tend to the severity of disease [75, 76].

## Diagnosis

As discussed above,based on various scientific study COVID-19 infection shares symptoms like normal flu, influenza infection, and other pneumonia type features which makes it difficult to diagnose with accuracy and sensitivity. Thus, WHO recommended molecular (e.g. PCR) testing of respiratory tract samples for the identification and laboratory confirmation of COVID-19 cases. Reverse transcription-polymerase chain reaction (RT-PCR) based molecular tests specifically detect viral RNA. RT-PCR is the only and early detection method available for SARS-COV-2 [86].

For the most sensitive detection of COVID-19, the collection and testing of both upper and lower respiratory samples include sputum and bronchoalveolar lavage fluid (BAL) is recommended while The US centers for disease control and prevention (CDC) recommends collecting only upper respiratory swab i.e. nasopharyngeal on priority as compared to the lower respiratory tract [19]. Before the testing procedure, specimen collection and processing are also considered a part of the diagnosis from the safety point of view. In RT-PCR based diagnosis, nucleic acid extraction is primarily required and specimens related to the COVID-19 infection must be processedin Biosafety level-2 (BSL-2) cabinet and transferred to the lysis buffer containing guanidinium based inactivating agents and non-denaturing detergent and then RNA extraction should be performed [64, 93].

As per the WHO, CDC and other research committees, RT-PCR diagnosis method of COVID-19 nucleic acid detection of nasopharyngeal (NP) and oropharyngeal (OP) swab sampling and further confirmation by next-generation sequencing is the best way to diagnose the COVID-19 infection [101]. The virus remains detectable in respiratory secretions for more than 1 month in some patients, but after 3 weeks cannot be recovered for culture due to entering a dormant phase. In the initial phase during the first week of post-infection, the virus may be detected in nasopharyngeal aspirates, throat swabs, and sputum samples, while in later phases viral RNA may be more easily detected in stool samples [169]. Besides, above expensive and poorly available and affordable in many developing countries for patients. The other methods of diagnosis like X-Rays, CT-Scan, and C-Reactive Protein (CRP) test has been adopted. In the regime of emergence of COVID-19 several recommendations of using these diagnoses with adequate supporting data has been appeared (Anonymous reviewer).

Myriad scientific research suggests that the presence of a virus depends on the days of the infection and varied slightly from person to person. Thus, the standard incubation time for the COVID-19 infected patient is 14–28 days. The incubation period of coronavirus disease (COVID-19) from public confirmed cases estimated by Lauer et al. [100]. Immunoassay has been developed for the rapid detection of COVID-19 infection such as antigen-based rapid diagnostic test and host antibodies detection test but WHO does not recommend these test over RT-PCR based diagnostic test due to lack of sufficient supportive data for these immunoassay test [200].

## Therapy and treatment: drugs and repurposing

There is no approved vaccines or therapies still exist for the disease. Worldwide, many of the companies working continuously for the development of vaccines or their kinds of drugs to fight with the COVID-19 infection. Development of vaccines for the COVID-19 is a quiet demanding course which is not a walk in the park.

Though S protein is currently considered to be one of the most promising targets for coronavirus vaccine development [223] and being considered for the development of vaccines to protect against MERS [4]. Supportive care is the only way to treat patients [154]. Further understanding of the structure and function of COVID-19 virus will provides elaborative knowledge on invasion and pathogenesis, to support the discovery of antiviral therapies and precise vaccine development.

Upon pathogen infection of the respiratory tract, the host immune system is activated to resist and clear the infection. Airway epithelium cells and alveolar macrophages release multiple pro-inflammatory cytokines and chemokines, such as tumor necrosis factor (TNF-α), interleukin-6 (IL-6), interferon (IFN) and other chemokines, including IL-8, monocyte chemoattractant protein-1 (MCP-1), and macrophage inflammatory protein (MIP). This release results in the attraction and activation of additional inflammatory cells, including macrophages and neutrophils, into the lungs, initiating the innate immune system that is crucial for the clearance and resolution of viral particles [82, 154]. Factors implicated in severe influenza include robust cytokine production, otherwise known as the “Cytokine storm”. Under physiological conditions, anti-inflammatory cytokines regulate the response of inflammation and attainment of equilibrium. However, the double-sided functions of cytokines could either be beneficial or detrimental to hosts. Under pathological conditions in which the balance is disrupted, pro-inflammatory responses may spiral out of control and excessive pro-inflammatory cytokines and inflammatory immune cells may contribute to additional tissue damage and inflammation [36, 130]. Also, Kumar et al. [92] carried out in silico identification of various drugs approved by the FDA and noted that protease-based drug-like small molecules can be used as potential inhibitors against SARS-CoV2.

### Herbal treatments

The effectiveness of herbal treatment to control the contagious disease has been reported vastly in 2003 during severe acute respiratory syndrome (SARS) outbreak [26, 221]. By the Chinese medicine system, the application of herbal treatment is mainly guided by the type of herb (based on the catalog of classic literature on herbs) and the patient’s symptoms or signs [221]. However, in the case of viral diseases, enough information related to directly targeting the viral cause is not predetermined, consequently; the herbal treatment is generally not guided by viral pathology. The presence of potent antiviral properties in various plants would be greatly important in viral diseases especially in COVID-19 and as per the need of the patient and knowledge about target sites of different natural components; various herbs have been investigated for co-therapy in COVID-19 case [195]. Some of the natural compounds have been screened and confirmed to directly inhibit these important proteins in SARS or Middle East respiratory syndrome (MERS) coronavirus [128, 129, 152, 168, 196, 222]. The herbal treatment, as an accurate and proved method with high accuracy in many countries has been used to aid treatment. For herbs that contains the same effective formulas as QC and HQC, Vit D and Zinc QC, or HQC + Vit D + Zinc+anticoagulant+cortisone+breath aid (such as with the nebulizers) have proved very high cure percentage of infections in early and mid-stages of COVID-19 (Anonymous reviewer). Various reviews on COVID-19 and immunity boosting with the use of herbs have appeared [55]. Immunomodulatory effects of medicinal plants such as Tulsi / *Ocimum sanctum*, *Cinnamomum zeylanicum*, *Zingiber officinale* and *Piper nigrum *know since ancient time and has also been accepted by science, when these plants bear medicinal and cultural values. These are stimulated our kitchen and influenced what we ate in different seasons and the remedies we used for common ailments. Their immune-modulatory, antiviral, antioxidant, anti-inflammatory, anti-platelet, anti-atherosclerotic, hepato-protective, reno-protective properties; seems to be effective in immuno-regulation for controlling viral infections like COVID-19 [55]. There are various anti-viral metabolites have been reported from various sources, which also effective in controlling COVID-19, to providing immunity to the host and other complex mechanisms. A myriad of metabolites of the same capacity has been enlisted here (Table 1). Further pre-clinical and clinical trials need to be done for the evaluation of safety and efficacy of this polyherbal formulation.

**Table 1:** Herbal constituents with possible mechanisms to combat SARS-CoV-2 (Adopted and updated from [75, 76])

Plant source	Active metabolite (s)	Possible target in SARS-CoV-2	Reference
Fruit and Vegetables	Quercetin	Inhibits 3CL^pro^ and interacts with viral HA protein to inhibit virus entry into the cell	Wu et al. [207]; Nguyen et al. [125]; Ryu et al. [151]
Green Chiretta; *Andrographis paniculate* (Acanthaceae)	Andrographolide	Inhibits 3CL^pro^ and virus-induced activation of RLRs signaling pathway	Yu et al. [216]; Enmozhi et al. [44]
Licorice	Glycyrrhizin	Inhibits replication, adsorption, and penetration of the virus	Cinatl et al. [29]
*Scutellariabaicalensis *Georgi.(Lamiaceae)	Baicalin	Inhibits 3CLpro and HIV-1 Env protein-mediated fusion with cells expressing CD4/CXCR4 or CD4/CCR5	Su et al. [171]; Li et al. [104]
Mint or deadnettle; *Patchouli* spp. (Lamiaceae)	*Patchouli* alcohol	Inhibits activation of PI3K/Akt and ERK/MAPK signaling pathways to block viral infection and replication	Yu et al. [217]
Vegetables and fruits such as celery, parsley, broccoli, onion leaves, carrots, peppers, cabbages, apple skins, and *Chrysanthemum* flowers	Luteolin	Inhibits 3CL^pro^ and the expression of the coat protein I complex and interferes with viral replication at an early stage of infection	Ryu et al. [151]; Yan et al. [212]
Citrus fruits such as oranges and tangerines	Hesperidin	Inhibits 3CL^pro^	Lin et al. [111]; Joshi et al. [85]
Rhubarb, Buckthorn, Aloe, Japanese knotweed and many species of fungi.	Emodin	Blocks the SARS-CoV spike protein and ACE2 interaction and inhibits 3a protein to reduces virus release	Ho et al. [71]; Schwarz et al. [160]
Grapes, wine, grape juice, peanuts, cocoa, and berries of *Vaccinium* spp.including blueberries, bilberries and cranberries	Resveratrol	Inhibits RNA and nucleocapsid expression	Lin et al. [112]
Grapes, tomatoes, broccoli, tea, and *Ginkgo biloba* leaves	Kaempferol	Inhibits 3a channel protein	Schwarz et al. [159]
Grains, nuts, seeds, vegetables, and drinks such as tea, coffee or wine	Lignan	Inhibits virus replication and 3CL^pro^	Wen et al. [196]
The bark of several species of plants principally the white birch	Betulinic acid	Inhibits virus replication and 3CL^pro^	Wen et al. [196]
*Salvia* spp.	Tanshinone	Inhibits 3CL^pro^ and PL^pro^	Park et al. [128]
The root of the Asian medicinal plant*Salvia* spp*.*	Cryptotanshinone	Inhibits 3CL^pro^ and PL^pro^	Park et al. [128]
*Salvia miltiorrhiza* Bunge (Chinese sage, red sage root, and the Chinese herbal;Danshen)	Dihydrotanshinone I	Inhibits 3CL^pro^ and PL^pro^ [128]	Park et al. [128]
*Salvia miltiorrhiza* Bunge (Chinese sage, red sage root, and the Chinese herbal;Danshen)	Tanshinone IIA	Inhibits 3CL^pro^ and PL^pro^	Park et al. [128]
Turmeric; *Curcuma longa* (Zingiberaceae)	Curcumin	Inhibits virus replication and 3CL^pro^	Wen et al. [196]
Died root of the *Lithospermumerythrorhizon (*Boraginaceae), *Alkannatinctorial* (Boraginaceae)	Shikonin	Inhibits 3CL^pro^	Jin et al. [84]
*Sophora* spp. (Fabaceae)	Matrine	Improves abnormal laboratory parameters and clinical symptoms inpatients, and significantly shortens the time to nucleic acid conversion	Yang et al. [213]

### Antiviral drugs

Lopinavir/ ritonavir (Kaletra®) is used in combination with other medicines to treat adults and children over 14 days of age who are infected with human immunodeficiency virus HIV-1 [170]. Lopinavir/ritonavir among SARS-CoV patients has been associated with substantial clinical benefits (fewer adverse clinical outcomes) [27]. As per the guidelines of the National Health Commission of the People’s Republic of China, the combination of lopinavir and ritonavir is currently a recommended antivirus regimen in the latest version of diagnosis and treatment of pneumonia caused by SARS-CoV-2. The Lopinavir & Ritonavir affect proteolysis in the coronavirus replication cycle (Handbook of International Pulmonologists consensus on COVID-19, 2020). Four patients with mild or severe SARS-CoV-2 pneumonia had been cured or have significant improvement in their respiratory symptoms after treatment with combined lopinavir/ritonavir (Kaletra®), arbidol, and Shufeng Jiedu Capsule (SFJDC, a traditional Chinese medicine) on the base of supportive care [191].Japan’s anti-flu drug Favipiravir developed by a subsidiary of Fujifilm showed considerable results in clinical trials over 340 patients (www.theguardian.com). They found it safe & effective in the treatment of COVID-19 patients. Further investigations are recommended in this context. Arbidol is an antiviral drug used to cure influenza in Russia and China found effective against SARS-CoV-2 at a concentration range of 10–30 μM in vitro.

**Fig. 4:**
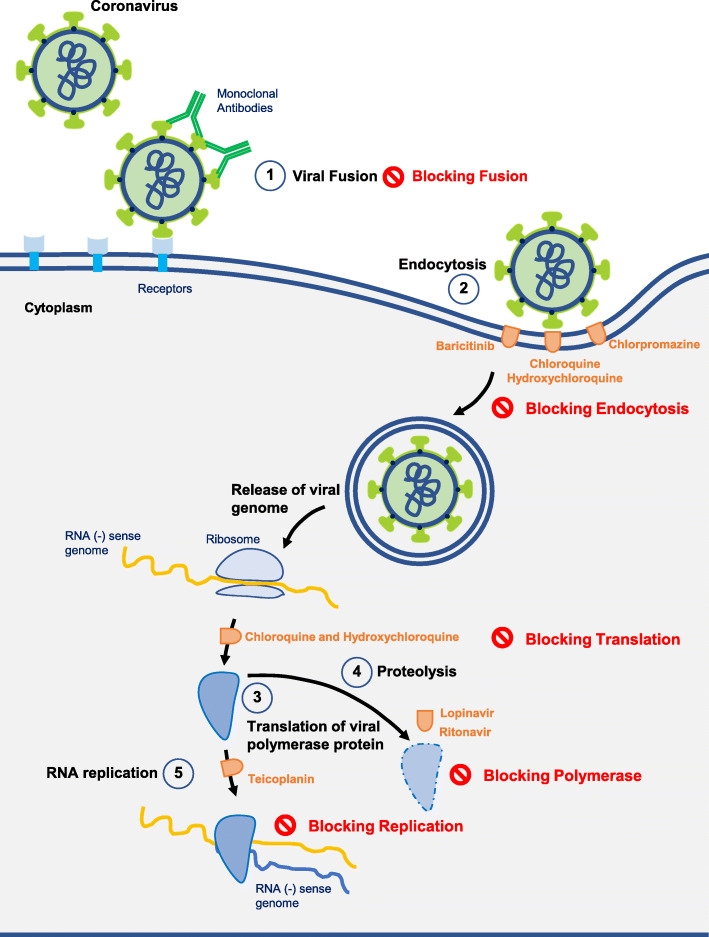
Possible drug targeting sites for the repurposing of different drugs in SARS-CoV-2 along with major stages of the life cycle in host cells. Major stages of SARS-CoV-2 life cycle in host cells and the probable site of action of different drugs

A study carried out on the discovery of new hydroxyethylamine analogs against 3CL^pro^ protein target of SARS-CoV-2 via molecular docking, molecular dynamics simulation, and structure-activity relationship studies in antiviral drug section established the importance of using antiviral drugs, as evidenced by suitability as a strong candidate for therapeutic discovery against COVID-19 [94]. The treatment of viral infection, the use of antiviral agents, and the specific vaccination is most appropriate, but now; the effective treatment for COVID-19 is available with some antiviral drugs only. The possible drug target with the major stages of the COVID-19 virus in host cells is shown in Fig. 4 to understand about the possible actions of viral inhibition in human host systems. The antiviral drugs targeting the viral proteins (linked with enzymatic activities or blocking viral replication mechanisms) or host proteins (engaged in the viral life cycle, regulating immune system functioning, regulators of cellular processes) have more potential as almost all the viruses encode proteins specifically polymerases for replication and transcription. The drugs exhibiting polymerase inhibition are of two types, a) allosteric inhibitors; b) nucleoside, and nucleotide substrate analogs. Nucleosides after phosphorylation to triphosphates by host cells are converted to active nucleotide and then act inhibitor by competing with natural nucleoside triphosphate and consequently, terminate the growth of viral nucleic acids.

### Remdesivir

Remdesivir, an antiviral agent initially used in Ebola virus clinical studies, revealed even more effective results against COVID-19 in vitro [191]. It is an adenosine analog, which incorporates into nascent viral RNA chains and results in premature termination. Further investigations of remdesivir are anticipated in human patients of COVID-19 on a priority basis. Although real-time therapy for COVID-19 is still in nutshell and not entirely any ‘magic bullet’ has introduced. In completed, ongoing and planned clinical trials, there are generic drugs promising therapies, prominently remdesivir [179]. Remdesivir is an analog of adenosine and is already known for the management of viral diseases. In the current scenario of this respiratory pandemic, remdesivir has overtaken on the other competitive drugs like hydroxychloroquine, chloroquine, lopinavir, and ritonavir in the context of its benefits over other lines of treatments [78, 127, 185]. In some of the trials, it has been observed that hydroxychloroquine and lopinavir or ritonavir showed little or no reduction in mortality of hospitalized COVID-19 patients, irrespective of standard care and management. However, yet in many countries, chloroquine or hydroxychloroquine being recommended as an option of COVID-19 treatment.

Remdesvir inhibits RNA-dependent-RNA-polymerase (RdRp) with its broad-spectrum antiviral activities against RNA viruses including SARS-CoV and MERS-CoV along with human CoV-229 E and CoV-OC43 replications [183]. Mentioned viral entities cause upper respiratory infection in children and its adults such as asthma, chronic obstructive pulmonary disease (COPD). Besides these, a member of δ coronavirus, pulmonary delta coronavirus (PDCoV) having the most divergent viral RdRp responsible for respiratory tract infections, which also treats using remdesivir [16]. The potency of remdesivir as an antiviral drug has also been proved by in silico test based on COVID-19’s RdRp built model [42]. Remdesevir selectively inhibits viral replication by targeting viral RdRp and inhibiting viral RNA synthesis followed by an efficient intracellular conversion into active triphosphate metabolites (NTPs). The RdRp amino acid sequences in SARS-CoV and MERS-CoV belonging to beta coronavirus of the B-linkages are up to 96% identical to SARS-CoV-2. Besides, MERS-CoV belonging to β coronavirus having C-linkages with only 71% identical with SARS-CoV-2 [59–61]. The effective inhibition of RdRp of MERS-CoV by remdesivir is a bit complexed that it acts with triphosphate (RDV-TP) as substrate and compete with its natural protagonist ATP. The incorporation of nucleotide analog is more efficient to make changes in growing RNA chains. The inhibitor RDV does not cause immediate termination of chain and it allows the addition of more nucleotide due to the presence of 3′-hydroxyl prime free to cause Poly A tails. Afterward, RNA synthesis is arrested at 3′ position [59–61]. Under in vitro studies, it is proved that remdesivir has the potentials to block SARS-CoV-2 even at a very low macromolecular concentration. On the other hand, in vivo model reflects a significant improvement in pulmonary pathology in MERS-CoV and SARS-CoV infected mice [161, 162].

In various clinical trials have been conducted on patients infected with SARS-CoV-2 with oxygen saturation below 94% (with/without oxygen support), the patients were treated with remdesivir (200 mg) intravenously for 10 days and from the first day (100 mg) daily over the next 9 days. The mortality rate during the study was found 18% among the patients receiving invasive ventilation and 5% among the patients not receiving invasive ventilation. During the follow up of the therapy, 68% of patients displayed an improved oxygen maintenance class. It was also observed that the risk of death was significantly increased in patients aged 70 years or older and among the patients with higher serum creatinine at baseline. 68% of patients showed clinical improvement and the risk ratio for patients receiving invasive oxygen in comparison to 2.78% of patients receiving non-invasive oxygen [62]. In another clinical trial, the results were clearer that the patients receiving remdesivir become recovered sooner than those receiving a placebo [7]. The European Medicines Agency recommends remdesivir positively for the treatment of SARS-CoV-2 [202]. However, in some cases, similar results have not been reported [194].

Grein et al. [62] noted the adverse effect during the compassionate use of remdesivir in patients with COVID-19. The symptoms include diarrhea, rash hypotension, abnormal liver function, and renal impairment. In 23% of patients of the study, serious adverse events like as acute kidney injury, septic shock, multi-organ failure were diagnosed. 8% of the patients discontinued the therapy due to severe adverse effects. The serious adverse events like acute kidney injury, septic shock, and multi-organ failure were observed in 8% of patients. According to another study reported by Wang et al. [194], the serious adverse effects were observed in 18% vs. 26% in remdesivir vs. control arm respectively; among remdesivir group, discontinued remdesivir (12%) compared to control (5%) was due to acute respiratory distress syndrome or respiratory failure in 5% patients of remdesivir group. In another clinical trial, the convalescent plasma therapy along with remdesivir has been proved beneficial in clinical trials [3]. Some precautions are required during the administration of remdesivir and no other evidence has been reported irrespective of nephrotoxicity. Remdesivir solution and lyophilized preparation (150 mg) contains 4.5–9.0 g expedient of sulfo-butyl-ether-β-cyclodextrin-sodium (SBECD). SBCED clears through the renal system and in some cases may cause moderate to severe renal impairment varying with individuals. In patients having glomerular filtration rate (GFR) ≥50% from baseline, close observation is required during remdesivir administration.

In the present scenario, no dose modifications of remdesivir have been recommended for patients suffering from mild and moderate renal impairment with COVID-19 besides causing severe renal impairment (eGFR< 30 ml/min) as a subside effect. Remdesivir cleaves by hydrolases and affects hepatic impairment is reported but few; reported to have subsided effects as influence alanine transferase (ALT) in patients with the upper limit of normal or severe hepatic function. No adverse effects on embryo-fetal development have been reported in a pregnant animal administered with remdesivir and on male fertility too. In COVID-19, the current recommendation of remdesivir doses is a bolus dose of 200 mg IV/ diluted in normal saline (0.9%) or 5% dextrose to be given over 60 min on day 1 followed by 100 mg IV to be given as diluted over 60 min for the next 5 days [166].

### Chloroquine and hydroxychloroquine

The chemical structure of chloroquine (CQ) and hydroxychloroquine (HCQ) are closely related. CQ and HCQ have a chiral center, which produces two enantiomers R(−) or S(+) forms or isomers [80]. Most clinically used CQ and HCQ exist as a racemic mixture (50:50) of both isomers which complicates the understanding of their PK and associated toxicity as they could behave differently inside the body [15, 53, 80, 153]. Several studies have revealed that both drugs have antiviral activity in vitro through different mechanisms [79, 120, 164]. Different studies have reported the ability of CQ to inhibit viral entry [39, 58, 147], uncoating [12], assembly, and budding [49, 156]. One of the suggested mechanisms by which CQ can affect the entry step of viruses is by inhibiting quinone reductase-2 [96] and this enzyme is required for the biosynthesis of sialic acid [187]. Sialic acid is involved in virus attachment and entry into host cells by several viruses including HCoV-OC43 and MERS-CoV [107, 181]. CQ potently inhibits the entry of SARS-CoV into cells by interfering with the glycosylation of its cellular receptor angiotensin-converting enzyme 2 receptor (ACE2). SARS-CoV-2 also uses ACE2 as a receptor for cell entry and it suggests a possible similar effect of CQ on SARS-CoV-2 at this step of virus replication [72]. In the early stages, CQ can also affect virus replication by inhibiting virus-endosome fusionlikely via increasing endosomal pH [89]. CoVs such as SARS-CoV can enter target cells via a pH-dependent mechanism in which the acidic pH of the lysosome facilitates the fusion of the viral and endosomal membranes resulting in viral particle uncoating and subsequent release of viral nucleic acid into the cytoplasm [214]. Besides this, CQ can also impair post-translational modifications of viral proteins through interfering with proteolytic processes [143] and inhibition of glycosylation via specific interactions with sugarmodifying enzymes or glycosyltransferases [157]. Moreover, it has been suggested that CQ could affect the cytotoxic mechanisms and works as an anti-autophagy agent in vitro [57]. CQ also works as an anti-inflammatory agent through reducing tumor necrosis factor (TNFα) release and suppressing TNF receptors on monocytes [37, 157]. HCQ has a similar effect to CQ in interfering with the glycosylation of ACE2, blocking virus/cell fusion, and inhibiting lysosomal activity by increasing pH [106].

HCQ can also impede major histocompatibility complex (MCH) class II expression which inhibits T cell activation, expression of CD145, and cytokines release [114, 184, 206]. HCQ has been shown to impair Toll-like receptors (TLRs) signaling through increasing endosomal pH and interfering with TLR7 and TLR9 binding to their DNA/RNA ligands thereby inhibiting transcription of pro-inflammatory genes [47, 67, 95]. All the above said mechanisms including immunomodulatory effects of CQ and HCQ have raised the interest in using these drugs in COVID-19 patients at risk of cytokines release syndrome (CRS) [226]. It has also been studied that afteroral administration of CQ and HCQ, their bioavailability can reach up to 80% with plasma peak time around 2–4 h [65, 80, 178]. Thus, parenteral administration, if available, might be a better route especially that oral administration has shown huge inter-patient variability [14, 65, 177]. The long half-life of both CQ and HCQ (range from 30 to 60 days) is likely attributed to their large volume of distribution (200 to 800 L/kg) and extensive tissue uptake [6, 31, 41, 52, 74, 139, 197, 211].

In China 15 clinical trials were conducted to test the efficacy and safety of CQ or HCQ in the treatment of COVID-19, 8 of which were based on the administration of CQ, 6 were of HCQ, and another included both CQ and HCQ [220]. So far, in a clinical trial involving more than 100 patients, the chloroquine phosphate group showed efficacy in reducing the exacerbation of pneumonia, improving lung imaging findings, and increasing the negative rate of virus nucleic acid test. Given these findings, the Guidelines (version 6) for treatment of COVID-19 recommends for chloroquine phosphate administration by oral route at a dose of 500 mg (300 mg for chloroquine) for adults 2 times/ day (no more than 10 days) [40]. In a clinical trial based on Hydroxychloroquine’s therapeutic effect in COVID-19 in 20 patients after 1–2 days of HCQ treatment, clinical symptoms in all patients improved (NO: ChiCTR2000029559). After 5 days of HCQ treatment,19 patients improved on lung imaging findings. Besides, none of the mild patients had an exacerbation of disease in the HCQ group. Regarding safety, two of them had adverse reactions of mild rash and a slight headache, and the adverse reactions disappeared after adjusting the regimen. The results of this clinical trial confirmed the short-term efficacy of HCQ in the treatment of COVID-19, which can effectively improve lung imaging findings, promote a virus-negative conversion, and shorten the disease course. Although the number of cases in the HCQ group was relatively small, current data can provide insights for clinicians. The efficacy and safety of HCQ in the treatment of COVID-19 need to be confirmed in further preclinical and clinical trials (NO: ChiCTR2000029559). The dosing regimen of HCQ, CQ and others have also been investigated for COVID-19 as follows: Hydroxychloroquine-400 mg (BIDX 2 doses, then 200 mg BID for 5 days), Remdesivir-200 mg (IV loading dose, then 100 mg IV for 10 days), Oseltamivir-150 mg (BID for 5 days), Lopinavir- 400 mg (BID for 10 days) Ritonavir-100 mg (BID for 10 days) Ribavirin-2 g (loading dose, then 600 mg TID) [133]. A summary of the FDA [174] with the use of hydroxychloroquine and chloroquine to treat hospitalized patients with COVID-19 is now available. This includes reports of serious heart rhythm problems and other safety issues, including blood and lymph system disorders, kidney injuries, and liver problems and failure.

### Tocilizumab

The syndromes caused by COVID-19 have been characterized by excessive production of inflammatory cytokines (interleukin (IL)-6, IL-10, and tumor necrosis factor-alpha (TNF-α) [24, 75, 76, 134, 163]. Hence, anti-cytokine therapy has been suggested as a treatment of COVID-19, although its safety and efficacy in this population is yet to be established [119]. Tocilizumab, an immunosuppressive agent, has also been found to be effective in vivo in COVID- 19 patients in China [208]. Tocilizumab is an FDA-approved IL-6 receptor antagonist commonly used to treat CRS secondary to CAR (chimeric antigen receptor) T-cell therapy [51]. Tocilizumab is theorized to treat the CRS that can occur in COVID-19 patients like its use in cytokine release syndrome (CRS) secondary to chimeric antigen receptor (CAR) T-cell therapy [224]. In two cases of COVID-19-induced CRS with elevated IL-6 levels and progression to sHLH (cytopenias, hypertriglyceridemia, elevated ferritin & LDH, hypofibrinogenemia), the treatment with tocilizumab has been found effective [138]. Evidence suggests a deficient T-cell response underlies the severity of COVID-19. CD8 and CD4 T cell levels are decreased in COVID-19 patients and correlate with the severity of disease [22]. Moreover, diabetes, which is associated with T-cell mediated immunosuppression, is a risk factor for COVID-19 [138]. IL-6 promotes immature thymocyte differentiation into cytotoxic T-cells and is needed for B-cell production of IgM and IgG. Thus, elevated IL-6 levels may be a compensatory mechanism for an impaired viraldirected cytotoxic T-cell response. Thus, decreasing IL-6 levels in COVID-19 may promote increased viral replication if tocilizumab is used too early in the disease course. A retrospective study has been performed to observe the efficacy of tocilizumab in treating severe or critical COVID-19 patients. Along with the basic anti-virus treatment, TCZ 400 mg was applied once to 20 patients intravenously. Within a few days, 75.0% improved oxygenation,the fever returned to normal and other symptoms improved remarkably. Besides, the opacity lung lesion on CT scans absorbed in 90.5% patients and the percentage of peripheral lymphocytes returned to normal in 52.6% patients. Their data suggests TCZ might be an effective treatment in severe patients of COVID-19 [220].

However, there is currently no data from rigorously conducted clinical trials evaluating tocilizumab use in COVID-19. Many clinical trials are actively recruiting subjects to determine the safety and efficacy of tocilizumab in the treatment of severe COVID-19 pneumonia in adult patients (NCT04315480, NCT04317092, NCT04320615, ChiCTR2000029765) [138]. These clinical trials may further elucidate tocilizumab’s effects on COVID-19-induced organ failure and mortality and correlate these outcomes with inflammatory marker trends.

### Antibiotics

Although there is no role for antibiotics in the treatment of coronavirus infection, 58% of patients in Wuhan were started on antibiotics [63]. Further, the use of empiric antibiotics is endorsed by the WHO to cover bacterial superinfections. It can be efficacious to treat COVID-19 patients provided that adequate clinical trials are conducted. The macrolide antibiotic-Bafilomycin A1 can be a promising candidate to treat the novel COVID-19. It is an endo/lysosomal V-ATPase inhibitor which mainly interrupts the function of ACE2. The ACE2 is mainly involved in SARS-CoV and SARS-CoV-2 infection by acting as a viral receptor, by which it may stop the viral cycle at the emerging stage itself [190]. Nitazoxanide is used to treat parasitic infections which were also found to be effective in treating a wide range of viruses including human coronaviruses in vitro at very low concentrations. It selectively blocks the viral hemagglutinin intracellular trafficking and insertion of this protein into the host plasma membrane. One more antibiotic which was found to be effective in viral infections is Azithromycin (a macrolide antibacterial). The drug effectively inhibits the growth of the Zika virus and the Ebola virus in-vitro [13, 116]. The synergistic effects of azithromycin and hydroxychloroquine combination were also reported to treat COVID-19 patients [56].

The report finding highlights the efficiency of this combination in clearing viral nasopharyngeal carriage within a short time in Covid-19 patients when compared to patients receiving only hydroxychloroquine. The hydroxychloroquine increases the pH withinacidic organelles and inhibits the entry of the virus [30, 146], producing antiviral effect (explained earlier) while the exact anti-viral action of Azithromycin is not known. It may have immunomodulatory properties that might be beneficial in the treatment of pulmonary viral infections. The molecule may decrease the inflammatory responses and production of excessive cytokine accompanying viral infections. The immunomodulatory mechanisms may be due to decreasing chemotaxis of neutrophils to the lungs by inhibiting cytokines and the formation of reactive oxygen species [56, 87]. Worldwide, Hydroxychloroquine along with antivirals (Lopinavir & Ritonavir) are being used to treat COVID-19 patients.

### Anti-inflammatory agents

There is a dilemma of anti-inflammatory therapy, balancing the risk and benefit ratio is a critical issue. The main concern is that anti-inflammatory medications, such as corticosteroid may delay the elimination of viruses and increase the risk of secondary infection, especially in those with an impaired immune system. Secondly, biological agents targeting pro-inflammatory cytokines can only inhibit specific inflammatory factor, and thus may not be very effective in curbing the CS in COVID-19 in which other cytokines maybe significant. Thirdly, some anti-inflammation medication such as JAK inhibitors also block INF-a production, which is important in fighting viruses, and theoretically may not be suitable for the treatment of inflammatory CS caused by viruses as COVID-19.

#### Glucocorticoids

Numerous clinical studies have reported the efficacy of glucocorticoids in the treatment of coronavirus pneumonia (such as SARS and MERS) or influenza pneumonia, but no consensus has been reached. Timely usage of glucocorticoids could improve the early fever, promote absorption of pneumonia, and obtain better oxygenation. However, some studies didn’t show beneficial effects with glucocorticoid, or even adverse reactions or delayed virus clearance, leading to a reduction in disease severity [5, 70, 210]. At present, systemic glucocorticoid administration was empirically used for severe complications to suppress CS manifestations in patients with COVID-19, such as ARDS, acute heart injuries, acute kidney complications, and patients with higher D-dimer levels [21, 75, 76, 173]. Chen et al. [23] reported 19 (19%) patients were treated with glucocorticoids for 3–15 days (median 5; 3–7 days), and methylprednisolone (1–2 mg/kg per day) are recommended for patients with ARDS, for as short a duration of treatment as possible. Wang et al. [191] reported 44.9% of patients of COVID-19 were given glucocorticoid therapy and no effective outcomes were observed. Russell et al. [150] reported clinical evidence did not support corticosteroid treatment for COVID-19 lung injury.

#### JAK inhibitors

The ACE-2 is the receptors for novel coronavirus pneumonia (SARS-CoV-2) is a cell-surface protein widely existed on cells in the heart, kidney, blood vessels, especially lung AT2 alveolar epithelial cells. SARS-CoV-2could invade and enter cells through endocytosis. One of the known regulators of endocytosis is the AP2-associated protein kinase 1 (AAK1). AAK1 inhibitors can interrupt the passage of the virus into cells and can help prevent virus infections. Baricitinib, a JAK inhibitor as well as an AAK1 inhibitor, was suggested a possible candidate for the treatment of COVID-19, considering its relative safety and high affinity. Therapeutic dosage with either 2 mg or 4 mg once daily was enough to reach the plasma concentration of inhibition [98]. The biggest concern about JAK inhibitors is that it can inhibit a variety of inflammatory cytokines including INF-a, which plays an important role in curbing virus activity. Further clinical trials and detailed analysis are warranted to confirm their efficacy. To date, there are some registered clinical trials of JAK inhibitor: “Study for safety and efficacy of Jakotinib hydrochloride tablets in the treatment severe and acute exacerbation patients of novel coronavirus pneumonia (COVID-19)” (ChiCTR2000030170); “Severe novel coronavirus pneumonia (COVID-19) patients treated with ruxolitinib in combination with mesenchymal stem cells: a prospective, single-blind, randomized controlled clinical trial” (ChiCTR2000029580).

### Vitamin C

Ascorbic acid (Vitamin C) consists of antioxidant properties. It is not having a direct lethal effect on viruses, but it was reported that viral respiratory infections in human beings are affected by the levels of vitamin C [69]. When infection occurs, the cytokine surge caused by infection is activated and neutrophils get accumulated in the lungs, destroying alveolar capillaries. Early clinical studies have shown that vitamin C is having the potential to inhibit these processes. Combining Ascorbic acid with other drugs will be helpful for affected COVID-19 individuals [148].

### Baricitinib

Baricitinib is another drug that is used in the treatment of rheumatoid arthritis. It can be also used to treat novel coronavirus [145]. It might target the endocytosis process. The receptor that SARS-CoV-2 uses to infect lung cells might be ACE2, which are prone to viral infection. The AP2-associated protein kinase 1 (AAK1) might be one of the known regulators of endocytosis. Disruption of AAK1 might interrupt the passage of the virus into cells and the intracellular assembly of virus particles [115].

## Vaccine development

**Fig. 5:**
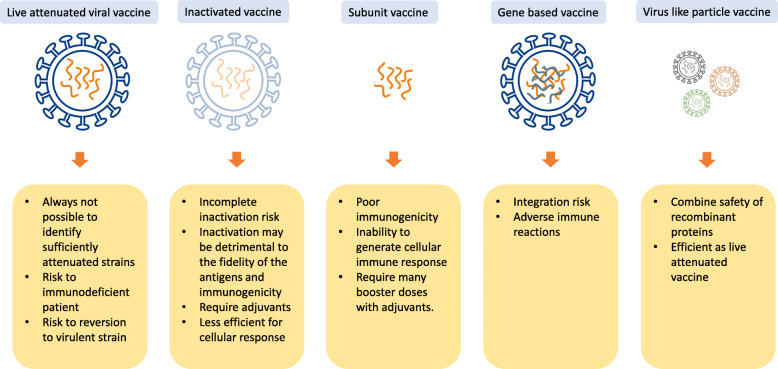
Possible types of viral vaccines can be developed for COVID-19

Vaccination is the most effective way to prevent influenza infection now. However, the high genetic variability of the virus renders the protection incomplete. In the case of the probability of the risk, some of the effective measures are to be adopted such as measurement or early detection, isolation and treatment of cases, as well as minimization of transmission through social interaction. The disease has no vaccine and no treatment in the recent scenario, forcing health authorities to resort to control tools dating back to the earliest days of empirical microbiology: isolation and quarantine. Many of the patients having strong immunity, can resist the lethality of the viruses and in the case of coronavirus, acquire immunity is delayed and therefore respiratory illness prevails before the immunity development and ability to survive to the infection. A viral vaccine can be developed by live-attenuated viruses, whole-killed viruses, split (proteins) viruses, recombinant subunits, virus-like particles etc. (Fig. 5). The scientist community is doing homework at full swing to create effective and safe vaccines. The efforts to develop a vaccine by various leading biotech companies, universities, and research agencies/institutions are underway. More than 90 and about reaching century vaccines are underway to be developed by various leading companies and universities across the world [17]. Scientists have trialed different methodologies, techniques, and efforts to a licensed their vaccines. At least six groups have already their human trials and started injecting formulations into volunteers’ arms under safety measures. As per the report published in July 2020 from the account of the ChAdOx1 nCoV-19 vaccine’sdeveloper, briefed about its immunogenicity and safety, as a result of phase 1 and 2. This way taking lead in amongst all runners world-wide [50]. In a vaccine race, a coronavirus vaccine shows lasting benefits in the people were front runner of COVID-19 vaccine trials, as they were reported to have high value of potent antibodies effective against coronavirus on and after the 4 months of their jab. Vaccine developed by Biotech from Moderna in Cambridge, Massachusetts has reported 94% effectiveness in their vaccine to cure COVID-19 [201].

## COVID-19 management

COVID-19 had become a pandemic, Globally, as of 5:02 pm CET, 11 December 2020, there have been 69,143,017 confirmed cases of COVID-19, including 1,576,516 deaths, reported to WHO (https://covid19.who.int/). The WHO and the United States Centre for Disease Control (CDC) have issued preliminary guidelines about diagnosis and disease control. But limited guidelines are available regarding the treatment of infected individuals due to lack of information about disease pathogenesis and non- availability of vaccine or specific anti-viral drugs for the treatment of the infected and critically ill patients. To control the COVID19 spread WHO, NIH and CDC and other scientific bodies releasing their guidelines, which can be concluded as, “Physical distancing with others is the best way to reduce the spread of coronavirus disease”. Primarily asymptomatic patients, with travel history or in contact with someone who has traveled recently are required to isolate themselves and undergo RT- PCR tests for Covid-19. The countries suffering from COVID19 banned social gatherings like in parks, marriages, restaurants, shops, and any other places [199]. Work from home culture was following by various bodies other than essential services. Self-isolation is an important measure taken by those who have COVID-19 symptoms to avoid infecting others in the community, including family members [106]. National Institutes of Health (NIH) published guidelines on prophylaxis use, testing, and management of patients with COVID19 based on scientific evidence and patient data. NIH appointed a panel to develop the guidelines for COVID19 control and treatment. Panel members include representatives from federal agencies, health care, and professional societies based on their clinical experience and expertise in patient management. Health care system declares step to step guidelines surpass management and prevention of COVID19 such as infection control guidance, protocols for using PPE, hand hygiene, discontinuing transmission-based precautions, post-mortem guidance and other guidance by facility type as alternate care sites, ambulatory care settings, assisted living facilities, pharmacies, and nursing home and long term care facilities. The spreadsheets & guidance release by health care units will help to maintain safety measures during the planning of the health care facilities and will optimize the transmission of coronavirus disease. Medical professionals caring for patients with coronavirus disease in 2019 are at high risk of contracting the infection [28].

It is inevitable to ensure health care professional safety not only to provide their services to patients but also to ensure the transmission of the virus to others [21]. World health organization has released guidelines for doctors and other health care professionals to deal with COVID19. They conducted training and webinars for physicians and nursing personnel on the management of patients with COVID19 and septic shock, ventilation strategy, management of aerosol-generating medical procedure, infection & prevention control practices, and psychological care of patients. Department of Health Research and ICMR recommended the use of hydroxychloroquine for prophylaxis of COVID 19 infection for the high-risk populations including all asymptomatic health care workers involved in the care of suspected or confirmed cases of COVID19. WHO guidance on specimen collection, processing, and laboratory testing is available [199]. The Panel recommends carefully monitoring, evaluating, and treating hospitalized patients with COVID19 for the incident and other serious events when indicated. Most people with COVID19 develop the only mild or uncomplicated illness, approximately 14% develop a severe disease that requires hospitalization and oxygen support, and 5% require admission to an intensive care unit. Patients with a mild clinical presentation (absence of viral pneumonia and hypoxia) may not initially require hospitalization, and many patients will be able to manage their illness at home. All findings suggested taking aggressive measures such as the use of masks, goggles, avoid social gatherings and follow other safety measures to ensure the safety during COVID19 outbreak especially when limited information about the transmission and infective potency of the virus is available.

## Conclusions

The very turn of the year 2020 took the whole world by storm as a novel Coronavirus first originated in the Wuhan town of China. In this continuation, a detailed and highly plausible package of current knowledge has been put forwarded in the form review demonstrating the virology, pathophysiology, genomics, and structure, and drug repurposes along with management of SARS-CoV-2.The increment of cases on a very fast move is providing a wider horizon to study biological interventions caused by SARS-CoV-2 in humans. We foresee the future emergence of novel virus variants or strains, to facilitate a sustainable life on the planet earth. Except for a few island nations, every country worldwide has been affected by the novel SARS-COV-2, thus declared as a pandemic. Since there is no FDA approved vaccine to COVID-19 by far, several alternative treatments and precautionary measures are explored to find a stable treatment for this disease. Most of these treatments and vaccines are assertive and based on the symptomatic suppression of the disease. In sum, we tried to provide key insights into SARS-CoV-2 and filling some gaps and uncertainties arise in certain points of this knowledge dissemination. It is clear more research still needs to be conducted to get a more detailed insight.

## Data Availability

All the data will be available whenever required.

## Abbreviations

SARS-Cov-2: Severe Acute Respiratory Syndrome Coronavirus
COVID-19: Corona Virus Disease
ACE-2: Angiotensin-converting enzyme 2
WHO: World health organization
CQ: Chloroquine
HCQ: Hydroxychloroquine
RT-PCR: Real Time- Polymerase Chain Reaction
SARS: Severe Acute Respiratory Syndrome
MERS: Middle East Respiratory disease
PL^pro^: Papain-like protease
3CL^pro^: 3C-like protease
HIV: Human Immunodeficiency Virus
CAR: Chimeric antigen receptor
RBD: Receptor-binding domain
RBM: Receptor-binding motif
HCoV: Human Corona Virus
TMPRSS: Priming response by host serine protease
ORF: Open reading frame
HE protein: Hemagglutinin-esterase
RNP: Ribonucleotide protein
RER: Rough endoplasmic reticulum
ERGIC: Endoplasmic reticulum-Golgi intermediate compartment
APC: Antigen presentation cells
CTLs: Cytotoxic T lymphocytes
MHC: Major histocompatibility complex
HLA: Human leukocyte antigen
RT-PCR: Reverse transcription-polymerase chain reaction
BAL: Bronchoalveolar lavage fluid
NP: Nasopharyngeal
OP: Oropharyngeal
TNF: Tumor necrosis factor
IL-6: Interleukin-6
IFN: Interferon
MCP-1: Monocyte chemoattractant protein-1
MIP: Macrophage inflammatory protein
FDA: Food and Drug Administration (USA)
SFJDC: Shufeng Jiedu Capsule
COPD: Chronic obstructive pulmonary disease
PDCoV: Pulmonary delta coronavirus
RdRp: RNA-dependent-RNA-polymerase
RDV-TP: Remdesiv triphosphate
SBECD: Sulfo-butyl-ether-β-cyclodextrin-sodium
GFR: Glomerular filtration rate
TLR: Toll-like receptors
CRS: Cytokines release syndrome
JAK: Jakotinib hydrochloride
AAK1: AP2-associated protein kinase 1
